# Use of radiolabelled choline as a pharmacodynamic marker for the signal transduction inhibitor geldanamycin

**DOI:** 10.1038/sj.bjc.6600558

**Published:** 2002-09-23

**Authors:** D Liu, O C Hutchinson, S Osman, P Price, P Workman, E O Aboagye

**Affiliations:** Cancer Research UK PET Oncology group, Department of Cancer Medicine, Imperial College of Science Technology and Medicine, Hammersmith Hospital, MRC Cyclotron Building, Du Cane Road, London W12 0NN, UK; MRC Cyclotron Unit, Hammersmith Hospital, Du Cane Road, London W12 0NN, UK; Cancer Research UK Centre for Cancer Therapeutics, Institute of Cancer Research, Royal Marsden Hospital, Sutton, Surrey, UK

**Keywords:** choline, ERK1/2, geldanamycin, phosphocholine, pharmacodynamic

## Abstract

There is an urgent need to develop non-invasive pharmacodynamic endpoints for the evaluation of new molecular therapeutics that inhibit signal transduction. We hypothesised that, when labelled appropriately, changes in choline kinetics could be used to assess geldanamycin pharmacodynamics, which involves inhibition of the HSP90 molecular chaperone→Raf1→Mitogenic Extracellular Kinase→Extracellular Signal-Regulated Kinase 1 and 2 signal transduction pathway. Towards identifying a potential pharmacodynamic marker response, we have studied radiolabelled choline metabolism in HT29 human colon carcinoma cells following treatment with geldanamycin. We studied the effects of geldanamycin, on net cellular accumulation of (methyl-^14^C)choline and (methyl-^14^C)phosphocholine production. In parallel experiments, the effects of geldanamycin on extracellular signal-regulated kinase 1 and 2 phosphorylation and cell viability were also assessed. Additional validation studies were carried out with the mitogenic extracellular kinase inhibitor U0126 as a positive control; a cyclin-dependent kinase-2 inhibitor roscovitine and the phosphatidylinositol 3-kinase inhibitor LY294002 as negative controls. Hemicholinium-3, an inhibitor of choline transport and choline kinase activity was included as an additional control. In exponentially growing HT29 cells, geldanamycin inhibited extracellular signal-regulated kinase 1 and 2 phosphorylation in a concentration- and time-dependent manner. These changes were associated with a reduction in (methyl-^14^C)choline uptake, (methyl-^14^C) phosphocholine production and cell viability. Brief exposure to U0126, suppressed phosphocholine production to the same extent as Hemicholinium-3. In contrast to geldanamycin and U0126, which act upstream of extracellular signal-regulated kinase 1 and 2, roscovitine and LY294002 failed to suppress phosphocholine production. Our results suggest that when labelled with carbon-11 isotope, (methyl-^11^C)choline may be a useful pharmacodynamic marker for the non-invasive evaluation of geldanamycin analogues.

*British Journal of Cancer* (2002) **87**, 783–789. doi:10.1038/sj.bjc.6600558 www.bjcancer.com

© 2002 Cancer Research UK

## 

There has been a recent shift in the paradigm for anticancer drug development, with a movement away from cytotoxic compounds towards agents that target specific gene products which create and drive the malignant phenotype (Garrett and Workman, 1999; Gelmon *et al*, 1999; Gibbs, 2000). Although some of these agents may result in reduction in tumour size, and may still be adequately evaluated by standard criteria based on tumour shrinkage or time to progression, most of these novel therapies may not be clinically assessable by conventional approaches (Garrett and Workman, 1999; Gelmon *et al*, 1999; Hudes, 1999).

There is, therefore, an urgent need to replace traditional response endpoints with more specific and relevant ones (Gelmon *et al*, 1999). Endpoints that measure effects on particular cell pathways, rather than general markers of tumour size or proliferation, may be particularly informative. However, developing such endpoints represents a significant challenge. Molecular events such as changes in phosphorylation and protein–protein interactions, are difficult to measure in the intact animal and patient.

The extracellular signal-regulated kinase (ERK) pathway operates downstream of activated receptor kinases and Ras, and regulates cell growth. This process involves activation of the cytoplasmic serine-threonine kinase Raf-1, which phosphorylates and activates mitogen activated kinase kinase mitogenic extracellular kinase (MEK), which in turn phosphorylates and activates ERK1/2 (p44/p42) on tyrosine and threonine residues. Phosphorylated ERKs shuttle into the nucleus where they activate transcription factors (Daum *et al*, 1994). To date, several inhibitors of the ERK cascade have been discovered (Gibbs, 2000), of particular interest to us. Geldanamycin is a benzoquinoid ansamycin antibiotic related to herbimycin A. Geldanamycin binds to the ATP binding domain of the heat shock protein 90 (HSP90), in a complex with various co-chaperones, leading to degradation of oncogenic client proteins such as Raf-1, p185ErbB2, cyclin-dependent kinase 4, some hormone receptors and also mutant p53 (Schulte *et al*, 1996, 1997; An *et al*, 1997; Grenert *et al*, 1997; Prodromou *et al*, 1997; Mayer and Bukau, 1999). Experiments with NIH3T3 cells have shown that Raf-1, for instance, exists in a native heterocomplex with HSP90, cdc37 and other proteins (Grenert *et al*, 1997). Inhibition of this complex by geldanamycin markedly decreases the cellular half-life of the Raf-1 protein, and this subsequently results in Raf-1 depletion and blockage of Raf-1-MEK-ERK signalling (Schulte *et al*, 1996, 1997). Currently, a geldanamycin analogue, 17-(allylamino)-17-demethoxygeldanamycin (17AAG) is undergoing Phase 1 clinical trials; therefore there is a need to develop a pharmodynamic endpoint to monitor the effect of such agents (Egorin *et al*, 1998; Kelland *et al*, 1999; Losiewicz *et al*, 1999; Clarke *et al*, 2000; Hostein *et al*, 2001).

Here we have studied the use of labelled-choline as a pharmodynamic marker of the geldanamycin-inhibited ERK pathway in a human colon carcinoma model. Choline is a marker for evaluating malignant transformation and proliferation by non-invasive methods such as magnetic resonance spectroscopy (MRS) and positron emission tomography (PET) (Smith *et al*, 1991; Negendank, 1992; Hara *et al*, 1997, 1998; Aboagye and Bhujwalla, 1999). After its transport into cells, choline is converted to phosphocholine by choline kinase in the presence of Mg^2+^ and ATP. This phosphorylation step commits extracellular choline to phosphatidylcholine biosynthesis and essentially ‘traps’ choline within the cell, making carbon-11-labelled choline an attractive PET probe. Phosphocholine and breakdown products of phosphatidylcholine are essential signalling molecules for cell growth, and phosphatidylcholine is the most abundant phospholipid constituent of the lipid bilayer in eukaryotic cells (Pelech and Vance, 1989; Cullis and Hope, 1991; Cuadrado *et al*, 1993; Exton, 1994). Despite its importance, little is known about the mechanisms that regulate choline kinase activity and hence phosphocholine production. A recent study on the yeast *Saccharomyces cerevisiae* revealed that choline kinase is activated through phosphorylation by the Ras-cyclic adenosine monophosphate (cAMP) pathway (Kim and Carman, 1999). Choline kinase has also been found to be a substrate for yeast protein kinase A (PKA) (Kim and Carman, 1999). Increased levels of phosphocholine are found in mouse fibroblast cell lines transformed by H-Ras, v-Src and Mos but not c-Fos (Ratnam and Kent, 1995; Hernandez-Alcoceba *et al*, 1997). Furthermore, transformation of benzo(a)pyrine-immortalised human mammary epithelial cells by ErbB2, led to increased phosphocholine levels (Aboagye and Bhujwalla, 1999). These findings suggest that ErbB2, Src, Ras and Mos may regulate choline kinase through the ERK pathway in mammalian cells.

Our results demonstrated that inhibition of ERK1/2 phosphorylation by geldanamycin is associated with a dose- and time-dependent inhibition of phosphocholine production. This specificity of the effect was verified by using the inhibitors U0126, roscovitine and LY294002. U0126 (1,4-diamino-2,3-dicyano-1,4-bis(2-aminophenylthio) butadiene) is a noncompetitive inhibitor of MEK with no other effects in the ERK cascade (Favata *et al*, 1998). Roscovitine is a potent and selective inhibitor of cyclin-dependent kinases, particularly cdk2 (Rudolph *et al*, 1996; Meijer *et al*, 1997). It acts by competing for the ATP binding domain of the cyclin-dependent kinases and has no significant effects on other kinases such as ERK1/2 at the concentrations used ( Rudolph *et al*, 1996; Meijer *et al*, 1997). LY294002 (2-(4-morphinyl)-8-phenyl-4H-1benzopyran-4-one) is a specific inhibitor of phosphatidylinositol 3-kinase (PI3K). It acts by competing for the ATP binding domain of the enzyme and has no effect on MAP kinase or ERK1/2 (Matter *et al*, 1994; Thomas *et al*, 1997; Muise-Hemericks *et al*, 1998). The results suggest that non-invasive imaging of phosphocholine formation may be a useful method for evaluating the effect of geldanamycin analogues *in vivo*.

## MATERIALS AND METHODS

### Cell culture

HT29 human colon carcinoma cells, MCF7 human mammary carcinoma cell (American Type Culture collection; Rockville, MD, USA) and MCF7-ADR human mammary carcinoma cells (obtained from Dr Zaver Bhujwalla, The Johns Hopkins University, Baltimore, USA) were cultured in RPMI 1640 medium (Sigma, Dorset, UK) supplemented with 0.4 mM L-glutamine, 20 U ml^−1^ penicillin, 20 μg ml^−1^ streptomycin and 10% foetal calf serum (FCS) (Gibco BRL Life Technologies, Renfrewshire, UK). Cells were incubated at 37°C (in 5% CO_2_ and at 100% humidity) and treated upon reaching approximately 50% confluency.

### Drug treatment

To determine the time-dependent effect of geldanamycin (Sigma, Dorset, UK) on ERK1/2 phosphorylation, cells were cultured in 90 mm Petri-dishes and treated with 2 μM geldanamycin for 2, 6, and 24 h. Cells were also incubated for 24 h, washed and incubated with drug-free medium for a further 24 h to evaluate recovery of activity. To determine the concentration-dependent effect of geldanamycin on ERK1/2 phosphorylation, cells were treated with geldanamycin (0.01, 0.2, 0.5 and 2 μM) for 24 h. To determine whether geldanamycin suppressed cell viability, cells were seeded in 96 well plates and treated with geldanamycin (0.01, 0.2, 0.5 and 2 μM) for 4, 24, 48 and 72 h. Similarly, the effect of geldanamycin on (methyl-^14^C)choline uptake by cells growing in 12-well plates was determined at the same concentrations for 1, 2, 4, 24 and 48 h. The effect of geldanamycin on (methyl-^14^C)phosphocholine production was determined by incubating cells, growing in 90 mm Petri-dishes, for 24 h with 0.01, 0.2, 0.5 and 2 μM geldanamycin. To further understand the role of the ERK pathway on phosphocholine production, validation studies were performed with other inhibitors including 1 mM hemicholinium (Sigma), 100 μM U0126 (Calbiochem Ltd., Nottingham, UK), 10 μM roscovitine (Calbiochem) and 5 μM LY294002 (Sigma). These concentrations of the inhibitors used have been reported previously to be efficacious (Hernandez-Alcoceba *et al*, 1997; Rudolph *et al*, 1996; Schulte *et al*, 1996; An *et al*, 1997; Meijer *et al*, 1997; Favata *et al*, 1998).

### Determination of protein content

Supernatants of homogenates obtained from treated and untreated cells were kept at 4°C prior to the protein assay. Protein concentrations were determined by a BioRad protein assay kit (Biorad, Hemel Hampstead, UK) which measures the absorbance at 595 nm of an acidic solution of coomassie blue-protein complex (Spector, 1978). Standard concentrations of bovine albumin were used to obtain a calibration curve.

### Western blot analysis

At the end of drug incubation, ERK protein phosphorylation was induced by treating cells with 100 nM of phorbol 12-myristate 13-acetate (PMA) for 10 min. After three washes with ice cold PBS, cells were lysed in TNES buffer (50 mM Tris-HCl, pH 7.5; 1% nonylphenoxyethanol (NP40); 2 mM ethylene diamine tetraacetic acid; 100 mM NaCl, 1 mM sodium orthovanadate; 1 mM phenyl methyl sulphonyl fluoride (PMSF); 25 mM NaF; and 25 mM β-glycerophosphate) for 1 h. The protein content of the lysates was determined by the BioRad method described above. Aliquots (containing 30 μg of protein) were loaded into each lane of a 10% polyacrylamide gel and eluted. Separated proteins were electro-transferred onto nitrocellulose membranes and incubated with 5% non-fat milk powder in TBS (10 mM Tris-HCl, pH 7.4; 0.9% NaCl) supplemented with 0.05% Tween 20 for 45 min to block non-specific interaction with antibody. The membranes were then incubated with a 1 : 5000 dilution of either rabbit anti-ERK (total ERK) or anti-phosphoERK polyclonal antibodies (Promega, Southampton, UK). The reaction was carried out overnight, followed by a further 1 h incubation with goat anti-rabbit antibody conjugated to horseradish peroxidase (Sigma) at 1 : 20 000 dilution. After three washes, bands were visualised using the ECL method (Nycomed Amersham plc, Buckinghamshire, UK).

### Cell viability assay

Relative cell viability (that reflects survival and proliferation) was measured by a colorimetric assay for determining the number of viable cells. At the end of drug treatment, the plates were washed twice and incubated at 37°C for 90 min with medium containing 20 μl of CellTiter 96®AQ_ueous_ One Solution Reagent (Promega). This solution is comprised of a tetrazolium compound and an electron-coupling reagent, phenazine ethosulphate. Reduction of the solution by growing cells leads to the formation of a water-soluble formazan derivative, which absorbs light at 490 nm (US Patent no. 5,185,450). Plates were vibrated for 2 min prior to reading their optical density in a scanning spectrophotometer (96-well plate reader; Anthos Labtech Instruments, Salzburg, Austria) at a wavelength of 492 and 620 nm (reference).

### Choline uptake assay

Following drug incubation, cells were washed twice and incubated for 1 h with (methyl-^14^C)choline chloride (Amersham Life Sciences Ltd., Rainham, UK) at 0.01 μCi well^−1^. After two washings with phosphate buffered saline (PBS), cells were detached by trypsinisation (0.5 ml, 5 min) and the action of trypsin neutralised with 0.5 ml of FCS enriched growth medium. Cells were then centrifuged (180 g, 5 min) and the cell pellets were re-suspended in 0.2 ml PBS and transferred into glass vials. Cells were solubilised by incubation with 0.8 ml of Soluene (Packard, Pangbourne, UK) at 50°C for 3 h. The radioactivity retained in cells was determined by scintillation counting after addition of 9 ml of Hionic fluor (Packard) to each glass vial. This assay measures the net accumulation of radioactivity following a 1 h pulse with radiolabelled choline.

### Phosphocholine production (whole-cell choline kinase assay)

Following drug incubation for 24 h (geldanamycin, roscovitine and LY294002) or 30 min (U0126), cells were washed twice with PBS to remove drug and incubated with medium containing (methyl-^14^C)choline chloride (Amersham) at 2 μCi per 7 cm^3^ flask. In the case of hemicholinium, radioactivity was added to drug containing medium at 30 min and incubated for 1 h. Cells were washed twice with PBS, then detached by trypsinisation (5 ml, 5 min) and the action of trypsin neutralised with 5 ml of FCS enriched growth medium. Cells were then centrifuged (3000 **g**, 10 min), resuspended in 2 ml of PBS and 3 ml of ice cold methanol, and then stored at −70°C for a minimum of 10 min in order to inactivate choline kinase. To release the intracellular (radioactively-labelled) metabolites, the cell suspensions were homogenised with a Polytron-Ultraturrax homogenizer (Jank and Kunkel KG, Breisgan, Germany) and centrifuged at 500 **g** for 10 min. Phosphocholine levels were analysed by high performance liquid chromatography (HPLC) with radiochemical detection using a μBondapak C18 column (7.8×300 mm, 10 μm; Waters, Watford, UK) and a mobile phase comprising of 1.5 mM K_2_HPO_4_ and 5 mM tetrabutylammonium hydrogen sulphate, pH 7.0. The flow rate of mobile phase and scintillant (Monoflow 2; National Diagnostics, Atlanta, GA, USA) were 2 and 6 ml min^−1^, respectively. Aliquots (50 μl) of (methyl-^14^C)betaine (prepared by oxidation of (methyl-^14^C)choline) were used as internal standard per ml of supernatant. Dilutions of (methyl-^14^C) phosphocholine (Amersham) spiked with (methyl-^14^C)betaine were used for calibration. Three to five samples were analysed for each dose level and at each time point. A set of samples was also eluted without the internal standard to ensure that no (methyl-^14^C)betaine peak was present in the samples. Phosphocholine levels (pmol μg^−1^ protein) were derived by converting the radioactivity associated with the phosphocholine produced to concentration units using the specific activity of phosphocholine. Protein concentrations of aliquots of the homogenate (50 μl) were determined as described above to enable normalisation of phosphocholine production to protein content.

### Statistical analysis

Statistical analysis of the data was performed using StatView version 1.04, 1991 (Abacus Concepts Inc., Berkeley, CA, USA). The statistical significance of differences in choline uptake, choline kinase activity and cell viability was determined using the Mann–Whitney *U*-test (two-tailed). *P* values ⩽0.05 were considered to be significant.

## RESULTS

### Suppression of ERK1/2 phosphorylation

**Figure 1:**
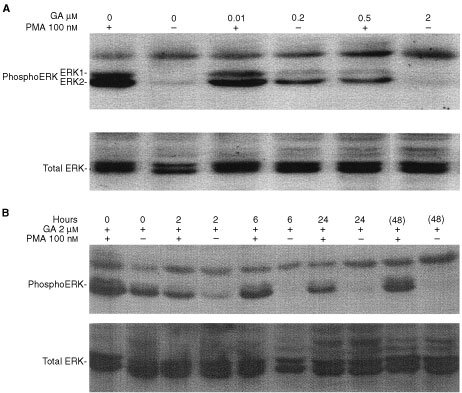
Western blots showing PMA-induced and uninduced phosphoERK1/2 and total ERK protein levels. (**A**) Treatment with different concentrations of geldanamycin (GA) for 24 h. (**B**) Treatment with 2 μM GA for different lengths of time. At 24 h cells were washed and placed in fresh media for a further 24 h (48 h).

We monitored the effect of geldanamycin on PMA-induced ERK1/2 phosphorylation in HT29 colorectal cancer cells by Western blotting (Figure 1A,B

). PhosphoERK1/2 were detected as 44/42 kDa proteins. There was a concentration-dependent reduction in PMA-induced ERK-1/2 phosphorylation after 24 h treatment with geldanamycin (Figure 1A). Maximum inhibition of PMA-induced ERK1/2 phosphorylation was observed with 2 μM geldanamycin. With regards to kinetics, inhibition of PMA-induced ERK1/2 phosphorylation by 2 μM geldanamycin was apparent at 6 h and highest at 24 h (Figure 1B). PMA-induced phosphoERK1/2 were detected when cells were incubated with geldanamycin for 24 h, washed, and incubated for a further 24 h in drug free medium suggesting partial recovery of PMA-induced phosphoERK1/2 levels within 24 h. The assay was insensitive to detection of non-PMA-induced phosphoERK1/2 levels beyond 6 h post-treatment. The differences in band intensities between Figure 1A and B were due to longer film exposure for the latter (to allow detection of non-PMA-induced phosphoERK1/2). Overall, the changes in phosphoERK1/2 levels were not accompanied by changes in total ERK1/2 protein (Figure 1A,B), indicating that the effect of geldanamycin were at the level of ERK1/2 phosphorylation rather than depletion of the protein. When cells were incubated with 10–100 μM of the MEK inhibitor, U1026 for 30 min, there was complete inhibition of ERK phosphorylation (data not shown).

### Suppression of cell growth

**Figure 2:**
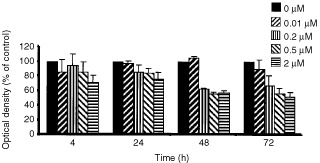
Inhibition of HT29 cell viability by geldanamycin (GA). Cells were incubated with drug from 4 to 72 h. At the end of the incubation, cells were washed and cell viability determined by a colorimetric assay as described in Materials and Methods section. There was a significant difference between untreated cells and cells treated for 4 h (*P*=0.016, 0.009 and 0.009 for 0.01, 0.2, 0.5 and 2 μM, respectively), 24 h (*P*=0.049, 0.049 and 0.015, for 0.2, 0.5 and 2 μM, respectively), 48 h (*P*=0.003, 0.004 and 0.0001 for 0.2, 0.5 and 2 μM, respectively), and 72 h (*P*=0.0001, 0.00003 and 0.000001 for 0.2, 0.5 and 2 μM, respectively). Error bars=1 s.d. (*n*=8–10).

Geldanamycin produced a concentration- and time-dependent decrease in viability of HT29 cells (Figure 2

). The percentage decrease in viability (compared to control) at a dose level of 2 μM was 25, 43 and 49% at 24, 48 and 72 h, respectively.

### Inhibition of choline uptake

**Figure 3:**
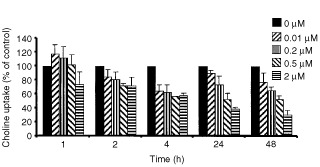
Effect of geldanamycin on the net accumulation of radiolabelled choline following a 1 h pulse in HT29 cells. Cells were treated with geldanamycin at the concentrations indicated and incubated for 1 to 48 h. Following drug treatment, cells were washed and (methyl-^14^C)choline uptake assessed as described in the Materials and Methods section. There was a significant difference between untreated cells and cells treated for 1 h (*P*=0.038 for 2 μM), 2 h (*P*=0.007 for 2 μM), 4 h (*P*=0.042, 0.008, 0.001 and 0.002 for 0.01, 0.2, 0.5 and 2 μM, respectively) and for 24 h (*P*=0.032, 0.013 and 0.003 for 0.2, 0.5 and 2 μM, respectively) and 48 h (*P*=0.0004 and 0.00005 for 0.5 and 2 μM, respectively). Error bars=1 s.d. (*n*=3–5).

The net accumulation of radiolabelled choline (choline uptake) is a function of both choline transport into cells and trapping through phosphorylation (by choline kinase). Figure 3

shows that the treatment of HT29 cells with geldanamycin produced a concentration- and time-dependent inhibition of (methyl-^14^C)choline uptake. Inhibition of (methyl-^14^C)choline uptake by geldanamycin was apparent at 2 h and maximal at 48 h post-treatment. After 4 h, doses as low as 0.01 μM produced a significant decrease in (methyl-^14^C)choline uptake (35% at 4 h; 33% at 48 h). The percentage decrease in (methyl-^14^C)choline uptake compared to control at a dose level of 2 μM was 61 and 70% at 24 and 48 h, respectively. As a positive control, 90 min incubation of HT29 cells with 1 mM of the choline transport and choline kinase inhibitor hemicholinium inhibited (methyl-^14^C)choline uptake by 89±2%. Combined with the cell viability measurements, these data indicate that the decline in choline accumulation is due in part to fewer cells rather than less accumulation per cell.

### Suppression of phosphocholine production

**Figure 4:**
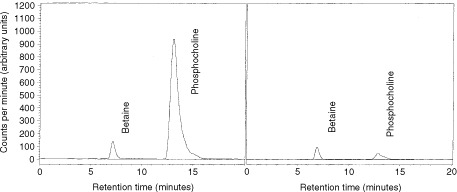
Representative chromatograms obtained from (**A**) untreated cells and (**B**) cells treated with U0126 (100 μM for 30 min). The chromatograms show a decrease in (methyl-^14^C)phosphocholine levels following treatment with U0126. (methyl-^14^C)Betaine was used as internal standard.

Choline kinase activity was evaluated by incubating cells with (methyl-^14^C)choline and measuring intracellular phosphocholine levels by a novel HPLC assay. (methyl-^14^C)betaine was used as internal standard after having shown that (methyl-^14^C)betaine was absent in these cells under identical conditions. Typical chromatograms are shown in Figure 4A,B

. Data were expressed as phosphocholine levels normalised to total cellular protein content (Table 1A

**Table 1:**
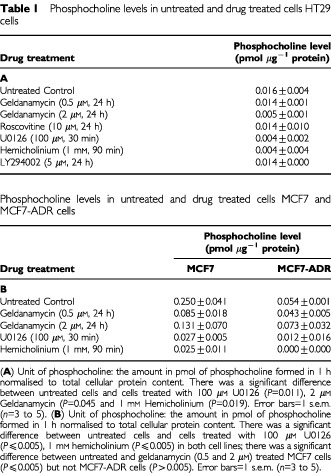
Phosphocholine levels in untreated and drug treated cells HT29 cells

).

Treatment of HT29 cells with geldanamycin produced a concentration-dependent decrease in phosphocholine production (Table 1A). At the highest concentration used (2 μM), the degree of inhibition was comparable to that seen with 1 mM hemicholinium. We hypothesised that choline kinase may lie immediately downstream of ERK. The MEK inhibitor, U0126, the cyclin dependent kinase 2 inhibitor, roscovitine and the PI3K inhibitor, LY294002 were used to probe the validity of this hypothesis. Brief (30 min) incubation of cells with 100 μM U0126 suppressed phosphocholine formation to the same extent as hemicholinium. In contrast, 24 h incubation with 10 μM roscovitine and 5 μM LY294002 had no effect on choline kinase activity. Phosphocholine production was also inhibited in MCF7 cells but not in the resistant MCF7-ADR cells (Table 1B).

## DISCUSSION

The focus of new drug development is now on abrogating the function of particular gene products important in driving the malignant phenotype and several agents are in preclinical and clinical development that target oncogenic signal transduction pathways (Garrett and Workman, 1999; Gibbs, 2000). Since traditional endpoints, such as tumour shrinkage and growth delay, may be insufficient or inappropriate for the objective assessment of such therapies, there is an urgent need for new surrogate endpoints to monitor pharmacodynamics during early clinical trials (Gelmon *et al*, 1999; Gibbs, 2000)

In this study, we have investigated the ability to monitor the pharmodynamic effect of geldanamycin, an inhibitor of HP90 and Raf-1, using carbon-14-labelled choline. All cells utilise extracellular choline (via bloodstream from food) as a precursor for the biosynthesis of the membrane phospholipid phosphatidylcholine (Zeisel, 1981). Phosphocholine is produced in cells via the action of choline kinase utilising ATP as the phosphate donor. This constitutes the major source of phosphocholine (see reviews by Pelech and Vance, 1989; Exton, 1994). The phosphocholine produced is trapped in cells as phosphocholine due to its charge, or is converted to phosphatidylcholine via cytidine diphosphate choline (see reviews by Pelech and Vance, 1989; Exton, 1994). Choline can be produced endogenously via the action of phospholipase D on phosphatidylcholine or from the hydrolysis of glycerophosphocholine and phosphocholine can be produced via the action of phospholipase C on phosphatidylcholine (Pelech and Vance, 1989). Due to the slow turnover of phosphatidylcholine, however, no cellular metabolites of (methyl-^14^C)choline, other than (methyl-^14^C)phosphocholine derived via the exogenous pathway, are detected in tumour cells after the ‘pulse time’ of 1 h used in this work (see also HPLC analysis). This makes radiolabelled choline an attractive tracer for monitoring this pathway. We have demonstrated that geldanamycin inhibited (methyl-^14^C)choline uptake at concentrations that inhibit Raf-1 dependent ERK1/2 phosphorylation and cell viability. The inhibition of (methyl-^14^C)choline uptake occurred earlier and at lower geldanamycin concentrations than those affecting ERK1/2 phosphorylation or cell viability. In HT29 cells, the geldanamycin analogue 17AAG that behaves similarly, was also shown to inhibit phosphoERK1/2 but not total ERK levels in a quantitative enzyme-linked immunosorbent assay (Wynne Aherne, personal communication). The change in (methyl-^11^C)choline uptake could be related to apoptosis, cell cycle arrest and/or direct inhibition of phosphocholine production. In future *in vivo* studies, assessment of the contribution of each of these processes will be important in understanding the mechanism of action of (methyl-^11^C)choline. Our results suggest that (methyl-^14^C)choline uptake is an especially sensitive measure of response to geldanamycin treatment. The cell viability studies demonstrated that the decrease in radiolabelled choline uptake was due at least in part to a decrease in cell viability. The *in vivo* application of this methodology may rest on the ability to detect trapping of choline in tumours as phosphocholine. Hence we have evaluated the effect of geldanamycin on phosphocholine production (normalised to protein content). Importantly, an association (but not dependence) between the decrease in choline uptake and inhibition of choline kinase activity in intact HT29 cells was demonstrated. A decrease in choline phosphorylation was not seen in MCF7-ADR cells at similar geldanamycin concentrations. These cells have been shown to be resistant to geldanamycin and the related ansamycin antibiotic herbimycin A (Benchekroun *et al*, 1994). Initial investigation of the mechanisms involved in the geldanamycin-induced decline in phosphocholine production utilised high concentrations of inhibitors of MEK (U0126), cdk2 (roscovitine), PI3K (LY294002) and choline transport (hemicholinium-3). Brief exposure of HT29 cells to U0126, under conditions, which do not lead to significant changes in viability, resulted in significant changes in phosphocholine production and ERK1/2 phosphorylation. We demonstrated that ERK1/2 phosphorylation and choline kinase activity could both be completely inhibited by both geldanamycin and U0126 (30 min incubation with 100 μM) but not by roscovitine (data not shown for ERK1/2 inhibited by U1026). From these observations one can infer that choline kinase regulation by this pathway lies downstream of MEK but upstream of cyclin dependent kinase/cyclin interactions. This is in keeping with previous transfection experiments with Ras, Src, and Mos, which act via phosphoERK1/2, as well as Fos, which acts downstream of phosphoERK1/2 (Hernandez-Alcoceba *et al*, 1997; Ratnam and Kent, 1995). In those studies, overexpression of Ras, Src and Mos, but not Fos, in mouse fibroblasts led to choline kinase activation. The inability of LY294002 to suppress phosphocholine production indicates that the PI3 Kinase pathway is not involved. Although results using inhibitors such as U0126 and geldanamycin suggest that ERK1/2 may be upstream of choline kinase, additional information, e.g., the influence of transfection with ERK or dominant negative ERK or MEK on choline metabolism will help strengthen the argument.

We have chosen to develop an endpoint for drugs, which act on the Raf-1-MEK-ERK signal transduction pathway. This is attractive for several reasons. One could potentially use (methyl-^11^C)choline-PET to select patients likely to respond to geldanamycin analogues. It remains to be seen whether (methyl-^11^C)choline could be used as a pharmacodynamic marker to monitor the effects of other inhibitors of the ERK cascade, including inhibitors of receptor kinases and Ras prenylation, (Gibbs, 2000). In the event of resistance arising during treatment with ERK pathway inhibitors, choline uptake studies should be able to detect this resistance phenotype, whereas a specific marker for say the receptor tyrosine kinase assay will not. Apart from these biological properties, (methyl-^11^C)choline is an attractive PET tracer since it is converted into phosphocholine, which is ‘trapped’ in cells.

The assay of (methyl-^11^C)choline by PET may be of immediate potential relevance in the clinical evaluation of the geldanamycin analogue, 17AAG. Unlike current invasive pharmacodynamic assays which measure Raf-1 depletion and HSP70 induction (O'Dwyer *et al*, 1999; Clarke *et al*, 2000), the measurement by (methyl-^11^C)choline does not require the biopsy sampling of tumours which can be problematic. Furthermore, necrosis, scar tissue formation and macrophage infiltration can confound assessment of response to cancer therapeutics by conventional anatomical imaging. Thus, a marker that is capable of detecting changes in proliferation and metabolism may be superior to conventional anatomical imaging. A potentially powerful application of this technology will be the ability to assess drug effects at concentrations consistent with maintained cell viability. The effects of U0126 on phosphocholine production (30 min treatment) and geldanamycin on (methyl-^11^C)choline uptake at early time points (a larger decrease in (methyl-^11^C)choline uptake compared to relative cell viability at 2 and 4 h), appear to support a potential role for radiolabelled choline in assessing inhibition of signal transduction at concentrations consistent with maintained cell viability. This hypothesis needs to be further tested. Geldanamycin treatment, however, inhibits growth of (or kills) HT29 cells and does not provide a very good model to fully assess cytostasis. *In vivo* studies to establish the pharmacokinetics and metabolism of (methyl-^11^C)choline will further support the usefulness of radiolabelled choline as a non-invasive pharmacodynamic marker.

In summary, we have shown that radiolabelled choline can measure the response of cells to geldanamycin treatment. Our results suggest that choline could be used as a non-invasive PET or MRS imaging probe to monitor the effects of geldanamycin analogues, which target the tyrosine kinase receptor-Ras-Raf-1-MEK-ERK cascade.
